# Clinical efficacy of azacitidine in the treatment of middle- and high-risk myelodysplastic syndrome in middle-aged and elderly patients: A retrospective study

**DOI:** 10.1515/med-2025-1151

**Published:** 2025-04-01

**Authors:** Liang Li, Jianjun Bian, Yuxuan Su, Chunchun Zhang

**Affiliations:** Department of Hematology, The Second Affiliated Hospital of Bengbu Medical University, No. 633 Longhua Road, Huaishang District, Bengbu City, 233000, China; Department of Hematology, The Second Affiliated Hospital of Bengbu Medical University, Bengbu City, 233000, China

**Keywords:** azacitidine, myelodysplastic syndromes, efficacy evaluation, retrospective study

## Abstract

**Objective:**

The aim of this study was to explore the efficacy and safety of azacitidine (AZA) in middle-aged and elderly patients with myelodysplastic syndrome (MDS).

**Methods:**

The clinical data of 59 middle-aged and elderly patients with middle- and high-risk MDS, who attended our hospital from April 2019 to January 2024, were retrospectively analyzed and were divided into an observation group (AZA) and a control group (conventional supportive treatment) according to the treatments, and the patients in the two groups were evaluated for their clinical efficacy and safety.

**Results:**

The overall response rate of the observation group was 66.67%. In terms of blood cells, the observation group’s blood cell level after four courses of treatment was significantly higher than that of the control group (*P* < 0.05). In terms of lactate dehydrogenase (LDH), the level of LDH decrease after four courses of treatment in the observation group was significantly better than that in the control group (*P* < 0.05). The safety of both groups was good.

**Conclusions:**

AZA is safe and effective in the treatment of high risk MDS in middle and old age patients.

## 1 Introduction

Myelodysplastic syndrome (MDS), a heterogeneous group of myeloid clonal disorders originating from hematopoietic stem cells, is characterized by abnormal myeloid cell development, manifested by ineffective hematopoiesis, refractory hematopenia, and high risk of transformation to acute myeloid leukemia (AML) [1]. MDS is a heterogeneous group of diseases, whose natural course is widely influenced by the degree of hematopenia, the percentage of myeloid primitive cells, and which present different prognoses [2]. The revised International Prognostic Scoring System (IPSS-R) is the most commonly used prognostic tool to predict the risk of leukemic transformation and overall survival [3]. Patients with low-risk MDS (LR-MDS) are primarily given supportive therapy and medications aimed at improving anemia [4]. While patients with high-risk MDS (HR-MDS) are treated with hypomethylating agents, azacitidine (AZA) or decitabine (DEC) [5].

Currently, in intermediate- and high-risk patients, the core objectives of treatment are timely disease control and prolonged survival, and AZA has a strong clinical profile in this regard. A systematic evaluation and meta-analysis conducted by Ken Hasegawa and colleagues [6] to assess the clinical outcomes of AZA monotherapy in patients with primary high-risk MDS showed that among the 16 included studies, the combined complete remission was 16%; partial remission was 6%; median overall survival was 16.4 months; and median duration of response was 10.1 months. The median time-to-response was 4.6 months, 10 and 30% of the adverse events were grade 3/4 anemia and thrombocytopenia, respectively, which demonstrated the clinical efficacy of AZA. However, the treatment of MDS is based on the prognostic grouping of MDS patients, as well as the comprehensive assessment of age, physical status, co-morbidities, and treatment adherence. Therefore, the efficacy of AZA treatment reported in different studies is not yet completely clear, especially in middle and old age MDS patients [7]. Since middle-aged and elderly patients are usually considered unsuitable for bone marrow transplantation, it is particularly important to clarify the advantages and disadvantages of their basic treatment modalities [8]. Therefore, we conducted a retrospective clinical study, aiming to investigate the efficacy of AZA monotherapy in middle-aged and elderly patients with high-risk MDS from a real-world perspective.

## 2 Materials and methods

### 2.1 Subjects of study

Eighty-nine cases of middle- and old-age MDS patients who attended the Department of Hematology of the Second Affiliated Hospital of Bengbu Medical University from April 2019 to January 2024 were reviewed, of which 75 cases were clearly diagnosed as medium- to high-risk patients. After collecting the general medical history data, efficacy evaluation data, and safety evaluation data involved in this study, 72 cases had complete data, and 1 telephone follow-up visit was made to the patients (or their families) to obtain informed consent, of which 9 patients were lost, and finally 59 patients were included in this study.

Inclusion criteria are as follows: (1) Middle and old age patients (≥45 years old) diagnosed with MDS, as defined by FAB (France-American-Britain) criteria [9]; (2) patients diagnosed as medium-high risk according to the International Prognostic Scoring System (IPSS-R) [3], (3) meet the criteria for demethylation therapy, without serious decline in liver, kidney, and lung functions and without serious cardiovascular and cerebrovascular diseases; (4) complete case information and informed consent.

Exclusion criteria are as follows: (1) Post-transplant recurrent or persistent MDS; life expectancy less than 12 months; (2) any coexisting major disease or organ failure; (3) HIV-positive or active viral hepatitis B or C; (4) acute uncontrolled infections and uncontrolled hemorrhage; (5) known or suspected allergy to AZA; (6) solid malignant tumors.

### 2.2 Research methodology

#### 2.2.1 Treatment

The observation group was given a single drug, AZA 75 mg/m^2^/day, subcutaneously injected at separate sites according to the dose used, and applied for 7 consecutive days, every 28 days as a course of treatment. Before proceeding to the next course of treatment, aspects such as adverse effects and hematological toxicity of the previous course of treatment were assessed to decide whether to reduce the dose of the drug or extend the waiting time for the next course of treatment, and the efficacy and the rate of adverse effects were assessed after four courses of treatment in order to decide on the subsequent course of treatment and dosage. In order to reflect the accuracy of the efficacy, the use of erythropoietin, androgens such as danazol and testosterone undecanoate, and immunomodulators such as lenalidomide or thalidomide is strictly prohibited for all patients under treatment. During treatment, supportive therapy such as blood transfusion and anti-infection treatment may be given depending on the patient’s condition.

The control group was given routine symptomatic supportive treatment, including androgens, such as danazol and testosterone undecanoate, immunomodulators, such as lenalidomide or thalidomide, and blood cell-boosting drugs, such as Diyu Shengbai tablets, Likejun, platelet-boosting capsule, and compound saponin pills. During the treatment, according to the patient’s condition, supportive treatment such as blood transfusion and anti-infection treatment can be given.

#### 2.2.2 Observation indicators

Efficacy assessment was determined according to the MDS International Working Group (IWG) efficacy criteria. Hematologic response assessment included complete response (CR), partial response (PR), hematologic improvement (HI), stable disease (SD), treatment failure, relapse after CR or PR, and progressive disease (PD) [10]. The overall response rate (ORR) included CR, PR, and HI.

All patients were regularly monitored for blood cells, lactate dehydrogenase (LDH), liver function, kidney function, and cardiac function before and after treatment. During the study period, response to treatment was assessed by regular blood tests (weekly for the first two cycles and every other week thereafter). Adverse events were summarized as the most serious level of severity and graded according to the Common Terminology Criteria for Adverse Events, version 4.0.

#### 2.2.3 Statistical methods

Data were processed with SPSS 23.0 software (IBM, USA, 64-bit). Measurement data that conformed to the normal distribution and variance chi-squared distribution were expressed as \[\bar{x}\pm s]\] ; comparison of consecutive time points was made by repeated measures ANOVA; two-by-two comparison of time points within a group was done by paired sample *t*-tests, and two-by-two comparison between groups was performed with independent sample t-tests; count data were expressed as *n* %; the chi-square test was used when *n* ≥ 40 and *T* ≥ 5; the Fisher exact probability method was used when *n* < 40 or at least one *T* < 1. A two-sided alpha level of 0.05 was considered to indicate statistical significance, with *P* values of less than 0.05.


**Informed consent:** Informed consent was obtained from all patients included in the study from themselves (or their families).
**Ethical approval:** The study was approved by the Ethics Committee of the Second Affiliated Hospital of Bengbu Medical University ([2024]KY008), and all procedures were conducted in accordance with the ethical standards of the 1964 Declaration of Helsinki and its subsequent amendments. The study was conducted based on retrospective patient data.

## 3 Research findings

### 3.1 Comparison of baseline characteristics

The baseline characteristics of patients are shown in Table 1, and the baseline characteristics between the two treatment groups were compared, which were found to be balanced and comparable (*P* > 0.05).

**Table 1:** Baseline characteristics of the two groups of patients

General information	Control group (*n* = 29)	Observation group (*n* = 30)	*χ* ^*2*^ or *t*-value/*P*-value
Sex (m/f)	19/10	22/8	0.425/0.514
Age (years, \[\overline{x}]\] ± s)	61.89 ± 5.29	60.47 ± 3.04	0.423/0.685
Body mass index (kg/m^2^, \[\overline{x}]\] ± s)	19.78 ± 3.24	20.16 ± 2.52	0.331/0.783
Normal karyotype (*n*, %)	21	25	0.268/0.605
**Cytogenetic risk by IPSS**			0.009/0.926
Intermediate	19	20	
High	10	10	
**Bleeding and infection on admission**			
Skin bleeding (*n*, %)	9 (31.03)	10 (31.25)	0.219/0.640
Bleeding gums (*n*, %)	8 (27.59)	9 (28.13)	0.194/0.713
Nosebleeds (*n*, %)	11 (37.93)	12 (37.50)	0.274/0.523
Gastrointestinal bleeding (*n*, %)	4 (13.79)	4 (12.50)	0.053/0.882
Urinary tract infections (*n*, %)	6 (20.69)	7 (21.88)	0.145/0.810
Respiratory infections (*n*, %)	16 (55.17)	18 (56.25)	0.269/0.60^#^

Note: “^#^” is the continuously corrected chi-square test.

### 3.2 Evaluation of clinical efficacy

After treatment, the ORR of the observation group was 66.67%, which was higher than that of the control group, which was 34.48% (*P* < 0.05), and it was better than that of the control group in CR, HI, PD, and the difference was statistically significant (*P* < 0.05). As for SD and PR, the difference was not statistically significant when comparing between the two groups (*P* > 0.05) (Table 2).

**Table 2:** Comparison of clinical outcomes of MDS patients in two groups

Clinical efficacy	Control group (*n* = 29)	Observation group (*n* = 30)
CR (*n*, %)	2 (6.90)	6 (20.00)^*^
PR (*n*, %)	5 (17.24)	5 (16.67)
HI (*n*, %)	3 (10.34)	9 (30.00)^*^
ORR (*n*, %)	10 (34.48)	20 (66.67)^*^
SD (*n*, %)	9 (31.30)	8(26.67)
PD (*n*, %)	10 (34.48)	2 (6.66)^*^

Note: Compared with the control group, ^*^ *P* < 0.05.

### 3.3 Blood cell evaluation

Before treatment, there were no statistically significant differences in blood cell levels between the two groups (*P* > 0.05). After treatment, the blood cell levels in both groups increased, and the differences between the groups were statistically significant (*P* < 0.05); within-group comparisons showed that the observation group performed better than the control group, with statistically significant differences (*P* < 0.05) (Table 3).

**Table 3:** Comparison of blood cells in two groups of MDS patients

Blood cell	Control group (*n* = 29)	Observation group (*n* = 30)
Pre-treatment PLT (×10^9^/L. *x̅* ± s)	12.13 ± 0.63	12.17 ± 0.72
Post-treatment PLT (×10^9^/L. *x̅* ± s)	23.53 ± 6.72^a^	92.46 ± 7.47^a*^
Pre-treatment N (×10^9^/L. *x̅* ± s)	0.67 ± 0.51	0.70 ± 0.43
After treatment N (×10^9^/L. *x̅* ± s)	1.01 ± 1.76^b^	4.03 ± 2.81^b*^
Pre-treatment Hb (g/L. *x̅* ± s)	32.70 ± 19.86	31.41 ± 20.71
Post-treatment Hb (g/L. *x̅* ±s)	45.80±17.53^c^	96.75 ± 21.56^c*^

Note: The superscript “a” indicates a statistically significant difference between groups with *P* < 0.05; the asterisk “*” indicates a statistically significant difference within groups with *P* < 0.05. PLT compared with our pre-treatment group, ^a^ *P* < 0.05; N compared with our pre-treatment group, ^b^ *P* < 0.05; Hb compared with our pre-treatment group, ^c^ *P* < 0.05; and ^*^ *P* < 0.05 compared with the same control group (platelets [PLT], neutrophils [N], and hemoglobin [Hb]).

### 3.4 Comparison of LDH before and after treatment

Before treatment, there was no statistically significant difference in LDH levels between the two groups (*P >* 0.05). After treatment, LDH levels in both groups decreased, with statistically significant differences observed when comparing before and after within each group (*P* < 0.05); when comparing between groups, the LDH levels in the observation group were lower than those in the control group, and this difference was statistically significant (*P* < 0.05) (Table 4).

**Table 4:** Comparison of LDH in two groups of MDS patients

LDH	Control group (*n* = 29)	Observation group (*n* = 30)
Pre-treatment (U/L. *x̅* ± s)	794.67 ± 20.63	795.58 ± 21.69
Post-treatment (U/L. *x̅* ± s)	329.43±18.63^a^	233.78±16.92^a*^

Note: ^a^ *P* < 0.05 compared with the group before treatment; ^*^ *P* < 0.05 compared with the control group in the same period.

### 3.5 Evaluation of non-hematologic adverse reactions

At the initial onset of the disease, both groups of patients had symptoms of bleeding and infection at multiple sites, and the difference between the groups was not statistically significant (Table 1). After the application of hemostatic drugs, platelet transfusion and anti-infection treatment during the period of demethylation and symptomatic supportive therapy to the two groups, the bleeding and infection symptoms of the two groups improved to a certain extent, and the difference between the two groups was found to be statistically significant when compared within the two groups (*P <* 0.05), and the difference between the two groups was not statistically significant in the comparison between the two groups (*P >* 0.05). No grade 3–4 adverse reactions occurred in both groups during the treatment period, and none of the patients discontinued the drug due to adverse reactions (Table 5).

**Table 5:** Comparison of non-hematologic adverse events in MDS patients in the two groups

Norm	Timing	Control group (*n* = 29)	Observation group (*n* = 30)
Skin bleeding (*n*, %)	Pre-treatment	9 (31.03)	10 (31.25)
	Post-treatment	2 (6.90)^a^	2 (6.25)^a*^
Bleeding gums (*n*, %)	Pre-treatment	8 (27.59)	9 (28.13)
	Post-treatment	3 (10.34)^a^	3 (9.38)^a*^
Nosebleed (*n*, %)	Pre-treatment	11 (37.93)	12 (37.50)
	Post-treatment	2 (6.90)^a^	2 (6.25)^a*^
Gastrointestinal bleeding (*n*, %)	Pre-treatment	4 (13.79)	4 (12.50)
	Post-treatment	0 (0.00)	0 (0.00)
Urinary tract infection (*n*, %)	Pre-treatment	6 (20.69)	7 (21.88)
	Post-treatment	0 (0.00)	0 (0.00)
Respiratory infections (*n*, %)	Pre-treatment	16 (55.17)	18 (56.25)
	Post-treatment	9 (31.03)^a^	10 (31.25)^a*^

Note: ^a^ *P* < 0.05 when compared with the group before treatment; ^*^ *P* < 0.05 when compared with the control group during the same time period.

## 4 Discussion

MDS is a clonal disease belonging to a heterogeneous group of hematopoietic stem cells, and because of the great variability in its natural course and prognosis, individualized regimen selection should be the mainstay of treatment [11]. Middle and old age patients have more comorbidities, and the functions of various organs of the body gradually decline with age. High-intensity treatment programs are likely to lead to more and severe complications, a decline in the quality of life of patients, an increase in the mortality rate, and a deviation from the therapeutic goal [12]. The quality of life of patients decreases, mortality increases, and the purpose of the treatment is deviated from the intended one. Although previous clinical studies have confirmed the advantageous role of AZA in the treatment of MDS, more detailed stratified studies are lacking. We therefore evaluated the clinical efficacy and safety of AZA in middle and old age patients with MDS in a retrospective study.

In this study, we evaluated the clinical efficacy and hematological performance of AZA in middle and old age patients with medium- and high-risk MDS, and the results showed that compared with conventional treatment, the ORR of the observation group was 66.67%, which was higher than that of the control group, which was 34.48% (*P* < 0.05), and the results of the observation group were better than those of the control group in terms of CR, PR, HI, SD, and PD, showing the good clinical efficacy of AZA. Meanwhile, the hematological assessment also showed that the blood cell level of the observation group was significantly higher than that of the control group after four courses of treatment (*P* > 0.05). This is consistent with the results of previous studies. Fenaux et al. [13] conducted a phase III, international, multicenter, controlled, parallel-group, open-label trial, in which patients with high-risk MDS were randomly assigned one-to-one to receive either the AZA (75 mg/m^2^/day for 7 days every 28 days) or conventional therapy (optimal supportive care, low-dose cytarabine, or intensified chemotherapy chosen by the investigators prior to randomization to the group) showed that at 2 years, 50.8% (95% CI: 42.1–58.8) of the patients in the AZA group survived compared with 26.2% in the conventional treatment group. This study established the better efficacy of AZA in high-risk MDS patients, and our study further showed that such advantageous efficacy coexisted in middle-aged and elderly patients, but as a retrospective study, limited by the nature of the study, the overall survival (OS) status of the patients was not obtained in this study, and it is worthwhile to further observe whether high ORR in this study can be converted into high OS. Meanwhile, systematic evaluation and meta-analysis also testified the results of this study [14]. A systematic evaluation and meta-analysis were conducted to analyze 11 trials involving a total of 1,392 MDS patients (DEC, *n* = 768; AZA, *n* = 624). The results showed that the pooled estimates of PR, HI, and ORR were significantly higher for AZA than for DEC, and further subgroup analyses showed that the advantages of AZA were more pronounced in elderly patients older than 75 years of age. The clinical advantages of AZA are related to its unique mechanism of action, and demethylation therapy is the mainstay of treatment for patients with MDS who are unable to undergo bone marrow transplantation. Demethylation therapy involves mainly administering DNA methylation transferase inhibitors to inhibit DNA methylation transferase, inducing tumor cell apoptosis, exerting anti-tumor effects, and promoting normal cell growth and development, so as to control the disease and prolong the survival period of MDS patients [15,16,17]. AZA is a class of pyrimidine nucleoside cytidine analogues, which can act on both DNA and RNA, influencing gene expression, DNA synthesis and metabolism, as well as cellular differentiation, exercise cytotoxicity, and give full play to the function of demethylation, thus improving the condition of the patient and increasing the survival rate [14,18]. It can improve the patient’s condition and increase the survival rate.

In this study, the safety of AZA in middle and old age patients at high risk of developing infections was assessed by the hematological profile of the patients and non-hematological adverse effects. The results showed that of the 30 patients in the observation group included in this study, 25 patients (83.3%) had infections at the time of onset, and of the 29 patients in the control group, 22 patients (75.9%) had infections, and there was no statistically significant difference in comparison, which showed that more infections existed in patients with MDS at the time of onset of the disease; at the same time, the majority of patients in the two groups had multi-site bleeding at the time of onset of the disease, which made the subsequent therapy which brought certain difficulties for the follow-up treatment. At the end of the observation group’s treatment, at the stage of myelosuppression, there were 12 patients (40%) who reached grade III–IV hematopenia, and there were 29 cases (96.7%) of secondary infections (including patients with infections at the time of the onset of the disease); at the same time, in the control group treatment, there were 24 cases (82.8%) of secondary infections (also including patients with infections at the time of onset of the disease), and the comparison showed no statistical significance. Most of them had bacterial and fungal double infections, with the site of infection mainly in the lungs, and one case of triple infection of bacteria, fungi, and herpes zoster virus was observed in the observation group, which showed that the infections might be more severe after demethylation treatment than the supportive treatment, but further observation is needed to clarify this. After active anti-infection, hemostasis, and platelet transfusion, infection control and bleeding improvement in both groups were significantly improved compared with the pre-treatment period, and adverse reactions such as interstitial pneumonitis and toxic encephalopathy appeared during the application of AZA [19].

Interestingly, this study also analyzed the prognostic impact of genetic mutations in the patients. In terms of genetic mutations, in the observation group, one case with mutations in SF3B1, ASXL1, RUNX1, and EZH2 at the time of initial diagnosis and one case with mutations in TET2 and ASXL1 were converted to acute myelogenous leukemia soon after treatment with demethylating therapy and had a poor prognosis, in line with the results of Venable et al.’s study [20]. Among the remaining high-risk patients in MDS, there were 8 patients (26.7%) with two or more gene mutations, and the efficacy assessment showed no patients with CR, 3 patients (10%) with PR, and 5 patients (16.7%) with HI, suggesting that despite the presence of two or more gene mutations, there can be a gain in some of the patients after AZA demethylation therapy. These results suggest that demethylation with AZA is also important for patients with mutations.

Also, the changes in LDH were evaluated in this study. Since MDS is characterized by increased hematopoietic inefficacy and bone marrow apoptosis and mitochondria are key regulators of apoptosis and sites of iron accumulation, which favor the production of reactive oxygen species (ROS) and adversely affect cell survival, energy metabolism may be an attractive therapeutic target [21,22]. Cilloni and colleagues have shown that energy metabolism may be an attractive therapeutic target in elderly MDS. de Swart and colleagues [23] showed that enhanced lactic acid fermentation was observed in elderly MDS subjects and suggested that this might be related to energy compensation. In the present study, serum LDH was measured at the initial onset of disease and was significantly elevated in both the observation and control groups; however, after demethylation and symptomatic supportive treatments, the values of LDH decreased in both groups compared to the time of onset of the disease, but the decrease was more pronounced in the experimental group. It shows that demethylation treatment has a real effect on the change of LDH, and with the subsequent treatment, the monitoring of LDH shows that the value of LDH is in the normal range in patients with continuous remission, while in patients with disease progression, the value shows a trend of continuous increase. In the future, we will continue to expand the sample size and extend the monitoring period to further evaluate the impact of this monitoring index on the prognosis of MDS patients.

In this study, we analyzed the case data of 59 patients and analyzed the clinical efficacy, hematological profile, and adverse effects of AZA monotherapy in middle-aged and elderly patients with medium- and high-risk MDS, confirming the good clinical efficacy and safety of AZA. However, this study was a single-center, retrospective study, which was limited by the study method, review time and follow-up, resulting in a limited study enrollment and a lack of better timeliness of the case data. Although we have controlled the generation of the abovementioned bias through a strict process, the study still suffers from limited extrapolation, and in the future we can use this study as the basis for larger-scale real-world studies in order to provide higher quality evidence.

## 5 Conclusions

MDS is a heterogeneous disease, and treatment should be individualized. Elderly patients often have multiple comorbidities, and high-intensity treatment regimens may lead to more complications and a higher mortality rate. As a demethylating agent, AZA inhibits DNA methyltransferase, promotes tumor cell apoptosis, has anti-tumor effects on MDS patients, and promotes the growth of normal cells, thereby controlling the disease and extending the survival. Additionally, the study analyzed the impact of genetic mutations on prognosis and found that some patients still benefit from AZA treatment even with multiple gene mutations. In the future, our team will expand the sample size and extend the monitoring period to further evaluate the impact of LDH monitoring indicators on the prognosis of MDS patients. The results of this study provide strong evidence for the use of AZA in middle-aged, elderly, and medium- to high-risk MDS patients. Future studies should be based on the results of this study and conduct larger-scale real-world studies to provide higher quality evidence. Overall, AZA monotherapy has shown significant efficacy and good safety in middle-aged, elderly, and medium- to high-risk MDS patients and can be considered as the preferred treatment option.
